# Lymph Nodes Evaluation in Rectal Cancer: Where Do We Stand and Future Perspective

**DOI:** 10.3390/jcm11092599

**Published:** 2022-05-05

**Authors:** Alessandra Borgheresi, Federica De Muzio, Andrea Agostini, Letizia Ottaviani, Alessandra Bruno, Vincenza Granata, Roberta Fusco, Ginevra Danti, Federica Flammia, Roberta Grassi, Francesca Grassi, Federico Bruno, Pierpaolo Palumbo, Antonio Barile, Vittorio Miele, Andrea Giovagnoni

**Affiliations:** 1 Department of Clinical, Special and Dental Sciences, University Politecnica delle Marche, 60121 Ancona, Italy; a.borgheresi@staff.univpm.it (A.B.); a.agostini@staff.univpm.it (A.A.); alessandrabruno92@gmail.com (A.B.); a.giovagnoni@staff.univpm.it (A.G.); 2 Department of Medicine and Health Sciences “V. Tiberio”, University of Molise, 86100 Campobasso, Italy; demuziofederica@gmail.com; 3 Department of Radiological Sciences, University Hospital Ospedali Riuniti, 60126 Ancona, Italy; letizia.ottaviani@ospedaliriuniti.marche.it; 4 Italian Society of Medical and Interventional Radiology (SIRM), SIRM Foundation, 20122 Milan, Italy; ginevra.danti@gmail.com (G.D.); roberta.grassi@policliniconapoli.it (R.G.); francescagrassi1996@gmail.com (F.G.); federico.bruno.1988@gmail.com (F.B.); palumbopierpaolo89@gmail.com (P.P.); vmiele@sirm.org (V.M.); 5 Division of Radiology, Istituto Nazionale Tumori IRCCS Fondazione Pascale IRCCS di Napoli, 80131 Naples, Italy; v.granata@istitutotumori.na.it; 6 Medical Oncology Division, Igea SpA, 80013 Napoli, Italy; 7 Department of Radiology, Azienda Ospedaliero-Universitaria Careggi, Largo Brambilla 3, 50134 Florence, Italy; federicaflammia@libero.it; 8 Division of Radiology, Università degli Studi della Campania Luigi Vanvitelli, 80128 Naples, Italy; 9 Department of Biotechnological and Applied Clinical Sciences, University of L’Aquila, 67100 L’Aquila, Italy; antonio.barile@univaq.it; 10 Abruzzo Health Unit 1, Department of Diagnostic Imaging, Area of Cardiovascular and Interventional Imaging, 67100 L’Aquila, Italy

**Keywords:** rectal cancer, nodal staging, magnetic resonance imaging, diffusion-weighted imaging

## Abstract

The assessment of nodal involvement in patients with rectal cancer (RC) is fundamental in disease management. Magnetic Resonance Imaging (MRI) is routinely used for local and nodal staging of RC by using morphological criteria. The actual dimensional and morphological criteria for nodal assessment present several limitations in terms of sensitivity and specificity. For these reasons, several different techniques, such as Diffusion Weighted Imaging (DWI), Intravoxel Incoherent Motion (IVIM), Diffusion Kurtosis Imaging (DKI), and Dynamic Contrast Enhancement (DCE) in MRI have been introduced but still not fully validated. Positron Emission Tomography (PET)/CT plays a pivotal role in the assessment of LNs; more recently PET/MRI has been introduced. The advantages and limitations of these imaging modalities will be provided in this narrative review. The second part of the review includes experimental techniques, such as iron-oxide particles (SPIO), and dual-energy CT (DECT). Radiomics analysis is an active field of research, and the evidence about LNs in RC will be discussed. The review also discusses the different recommendations between the European and North American guidelines for the evaluation of LNs in RC, from anatomical considerations to structured reporting.

## 1. Introduction

Rectal cancer (RC) is one of the leading causes of cancer-related deaths. The latest data from GLOBOCAN 2021 report RC as 8th among all cancers worldwide in terms of both incidence (3.9%) and mortality (3.2%), affecting 732.210 new individuals worldwide each year [1]. Despite the noticeable improvement in early diagnosis and management, morbidity and mortality remain high, with a 5-year survival of 64.7% [2]. One of the key prognostic factors for rectal cancer is the involvement of lymph nodes (LN) [3]; therefore, in these patients, preoperative neoadjuvant therapy is indicated to reduce the local recurrence rate [4]. 

Nodal involvement can be evaluated with different imaging methods, such as endoscopic ultrasound (EUS), computed tomography (CT), magnetic resonance imaging (MRI), and [18F] fluorodeoxyglucose-positron emission tomography (FDG-PET). Out of them, MRI has the highest contrast resolution for the soft tissues, allowing the best depiction of neoplastic lesions, their anatomical relationships, the depth of the rectal wall involvement, extramural venous invasion (EMVI), circumferential resection margins (CRM), and the assessment of the nodal (N) stage. For these reasons, MRI examination is considered the gold standard for locoregional staging and restaging in RC according to the main international guidelines [5,6,7]. However, MRI is less accurate in N staging than tumor (T) staging with values of sensitivity and specificity ranging between 58–77% and 62–74%, respectively [8,9,10]; other imaging modalities such as CT and EUS showed comparable diagnostic accuracy [11]. 

The reason for such diagnostic performances of radiological techniques relies on the use of mere dimensional criteria for the assessment of LN; however, 15% of LN ≤5 mm, reported as normal on MRI, can be metastatic [12,13]. Therefore, there is a consistent risk that some patients are under-staged and undergo surgical resection without neoadjuvant treatments, with an increased risk of recurrence and metastasis. Conversely, in patients with RC, 25% of LNs are over-staged [12] and potentially overtreated, with significant morbidity in the short (e.g., proctitis) and long period (e.g., genitourinary or anorectal dysfunctions), and significant influence on the quality of life [14]. Moreover, the American Joint Committee on Cancer (AJCC) included in the N-stage the entity of tumor deposits (TD): if TDs are detected, the stage N1c is assigned regardless of the presence of abnormal LN and the T-stage [15]. Therefore, LN evaluation is crucial for the staging of RC, and new tools are necessary to improve the diagnostic accuracy for an adequate treatment.

In this narrative review, we report the up-to-date evidence on the imaging techniques for the assessment of LNs in patients with RC. The review starts with the MRI protocol and the anatomical considerations about LN morphology and the different lymphatic drainages. From the routine MRI study, we will move to functional MRI techniques, such as diffusion-weighted imaging (DWI) and further evolutions (Intravoxel Incoherent Motion, IVIM, Diffusion Kurtosis imaging, DKI) and dynamic contrast-enhanced (DCE), which are gaining relevance in a clinical scenario where the role of FDG is still predominant. In each section, the main strengths and limitations of each technique will be discussed. After the techniques widely used in clinical routine, the use of Ultrasmall superparamagnetic iron oxide (USPIO) nanoparticles, the role of Dual Energy CT (DECT) and Radiomics will also be reported. The main differences between the European guidelines [16] and the North American Society of Abdominal Radiology [17] will be highlighted, including the different recommendations for structured reporting. 

## 2. MRI Protocol: The Basics

Both European and American guidelines agree that MRI examination for RC staging should be performed with a magnet ≥1.5 T with an external body coil covering from the aortic bifurcation to the anal verge [16,17]. As for other districts, the T2-weighted sequences (T2W) without fat suppression are preferred for the morphological evaluation of LN [18]. Therefore, in the recommended protocol for staging and restaging purposes, the assessment of regional LN is performed with a high-resolution 2D fast-spin-echo (FSE) T2W with a small field of view (FOV) oriented on the axial plane of the rectal lesion with a slice thickness ≤3 mm; the distant nodal stations are evaluated with a T2W FSE sequence including the entire pelvis on the axial plane from the aortic bifurcation to the anal sphincter [16,19,20]. 

## 3. Lymph Nodes and Tumor Deposits: Anatomical Considerations

A general principle in oncologic imaging is that the localization of the primary tumor suggests the most frequently involved nodal groups; thus, proper knowledge of lymphatic drainage is necessary for accurate tumor staging [21,22,23,24,25]. 

The lymphatic drainage of the rectum does not follow the arbitrary subdivision in three levels, i.e., lower (0–5 cm), middle (5–10 cm), and upper (10–15 cm) from the anal verge [4]. The rectal lymphatic vessels originate from the intramural lymphatic plexus and drain into the LNs of the mesorectum or the sigmoid mesocolon; these are named mesenteric nodes. The mesenteric lymphatics then go from mesenteric LNs to the retroperitoneal LNs and toward the pelvic sidewall, grossly following the rectal blood vessels that provide the names of each nodal group [25].

Usually, the most probably involved LN is located within a range of 1 cm proximally and distally to the tumor; the tumor subsequently disseminates cranially in >90% of cases [3,26,27,28]. The peritoneal reflection (PR), usually well-identifiable on MRI scans, represents the main anatomical landmark for different pathways of lymphatic spread of RC [29,30]. Tumors located above PR mainly drain into the mesenteric LNs and are associated with a higher risk of distant metastases [25,31]. Conversely, tumors originating, respectively, at the level or below the PR have a high likelihood of spreading to the nodal groups of the pelvic sidewall, the former with a probability of 21% and the latter up to 41.8% [25]. The lower is the position of the tumor, the higher is the probability of spread to the lateral LNs (see below), ranging from 11.4% for tumors between 4 and 6 cm from the anal verge to 33.3% of tumors below 4 cm [29,32]. 

The latest AJCC Tumor-Node-Metastasis classification (TNM 8th edition) defines the regional LNs: these include the mesorectal/pararectal, superior rectal, inferior mesenteric, internal iliac, and inferior rectal LNs without mentioning the obturator nodes; however, the obturator LNs are usually included within the regional LNs [15,33]. The LNs out of these groups are considered non-regional by the AJCC, and therefore as a distant spread or metastatic (M) disease. In the common terminology, external and internal iliac LNs, and obturator nodes, are referred to as “lateral lymph nodes”; however, while external iliac LNs are less commonly involved and should be considered as non-regional involvement (M1-stage), the internal iliac and obturator nodes are one of the primary sites of tumor spread and constitute a regional involvement (N-stage). Inguinal LNs are considered non-regional (M-stage) as well, except for distal RC extending below the dentate line, and therefore considered regional LN as in anal cancer (N-stage). A recent survey from Lambregts et al. pointed out that there is a knowledge gap between radiologists from different institutions on the precise anatomical landmarks of LN compartments; the authors proposed a standardized map based on a specific oblique-axial MRI acquisition, taking into account the surgical and radiotherapy definitions as well [22,34,35]. Table 1 summarizes the LN compartments and the anatomical landmarks included in the above-cited survey [34].

**Figure 1:**
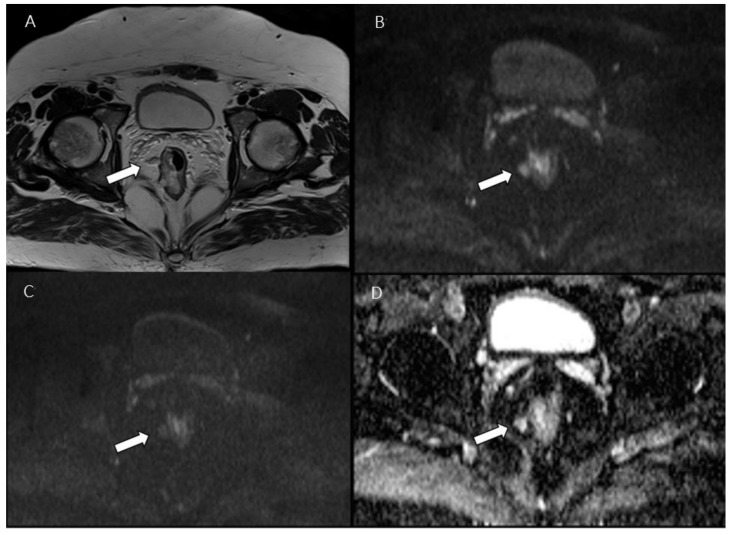
Man 53 y.o. with mucinous rectal cancer. Tumor deposit (arrow), assessed in T2W sequence (**A**), in b 50 s/mm^2^ (**B**), in b 800 s/mm^2^ (**C**) and ADC map (**D**).

The 8th AJCC also clarifies the definition of tumor deposits (TD) as the presence, within the lymphatic drainage area of RC, of a discrete tumor nodule without any identifiable continuity with neural, lymphatic, or vascular structures (Figure 1) [15,36]. TDs are considered as an independent pathway of tumor spread and have a negative prognostic value, being correlated with nodal metastases and EMVI [37,38]. Therefore, TDs should not be added to the total count of positive LNs; TDs upstage the RC to N1c regardless of the presence of abnormal LNs, automatically assigning a stage III [15]. However, there is raising consciousness about the inadequate integration of TD in the actual staging algorithm with suboptimal stratification of the risk; the COMET trial is designed to overcome these issues [34,38,39,40,41].

## 4. Nodal Assessment in Clinical Routine: Morphology

In oncological imaging, an involved LN is assumed to be increased in diameter. However, the application of a size threshold alone is unreliable in RC since LN increase may be caused by inflammatory/fibrotic processes [42,43]. Moreover, in RC there is an increasing awareness about the micro-metastases [15,44], which are associated with a worse prognosis [45]. It has been reported that about 30–50% of metastatic LN are ≤5 mm [13,31,42,46] and that micro-metastases can be present even in LNs less than 3 mm in diameter [9,44]. Moreover, a higher T stage has been correlated to a higher number of small, positive LN [31]. This is particularly relevant since 15% of positive LN < 3 mm were not detected at the preoperative MRI in a node-by-node comparison with pathology [9].

Therefore, in 2012 the experts’ panel of the European Society of Gastrointestinal and Abdominal Radiology (ESGAR) stated that no single-size threshold was sufficiently accurate to differentiate the benign from the metastatic LNs [47]. Thus, some studies proposed to add other morphological criteria beyond the diameter to increase the sensitivity and the specificity. Brown et al. were the first to combine the nodal profile and the signal intensity (SI) with dimensions, reporting an improvement in diagnostic performances of MRI for nodal staging in RC [12]. Subsequently, Kim et al. confirmed that indistinct or spiculated borders in addition to size can be used to predict the involvement of regional LN [48]. Recent studies on small cohorts proposed the interruption or absence of the chemical-shift artifact as an additional criterion for the assessment of nodal status [49,50].

Consequently, the 2016 ESGAR panel reiterated the low accuracy of dimensional measurements and proposed additional morphological criteria as beneficial for nodal assessment both for mesorectal and extra-mesorectal (obturator and iliac) LNs; therefore, the round shape, the irregular border, and/or the heterogeneous SI are considered suspicious of nodal involvement [16]. Recently, a large meta-analysis including only papers with a node-by-node comparison between pathology and MRI, confirmed that adding morphological features (e.g., irregular margin and mixed-SI) to size criteria improves the sensitivity and specificity of MRI; however, the relatively small diagnostic improvement was explained with the subjective assessment of these findings by the radiologists [10]. 

The heterogeneity of SI has been correlated to the presence of necrosis, extracellular mucin, or calcifications [12,51]. Nodal calcifications are a frequent finding in some rectal tumor subtypes such as mucinous adenocarcinomas: even if CT is the gold standard, MRI shows an acceptable diagnostic performance [52]. Chen et al. in a retrospective study compared the diagnostic accuracy of 2D-TSE T2W, 3D gradient echo (GRE) T1W, and CT for the detection of nodal calcifications [51]. Although 3D-GRE T1W is the most susceptible to field inhomogeneity, researchers observed a comparable specificity between the two MRI sequences. Moreover, on high-resolution MRI sequences, the area of signal drop appeared larger than the calcified area, with easier detection of malignant nodes even if compared to CT [51]. 

The ESGAR 2016 panel proposed the dimensional and morphological criteria for nodal assessment, being aware of the lack of evidence about the lateral LNs [35,53,54,55,56]. Following the publications of the ESGAR Guidelines, the Lateral Node Study Consortium performed a retrospective, multicenter study including cT3/4 low RC underwent surgical resection with curative intent [35]. The Consortium highlighted that the morphological features do not increase the diagnostic performance for the assessment of lateral LNs at primary staging; the cut-off of ≥7 mm can be applied to LNs only in the specific case of cT3/4 low tumors since they demonstrated a significantly higher risk of local recurrence [57]. 

MRI with dimensional criteria demonstrated higher sensitivity and specificity in the post chemoradiotherapy (CRT) setting than in primary LN staging. After CRT, most of the LNs become smaller or may disappear at MRI, therefore it can be assumed that LNs remaining visible after CRT are still at risk of involvement [58]. In this setting, unlike in the primary staging, the nodal size should accurately predict positive LNs with a cut-off of ≥5 mm regardless of other morphological criteria [59], while other studies proposed a lower threshold (≥2.5 mm) with negative predictive values of up to 95% for the identification of ypN0 patients [58,60]. Thus, even if the dimensional criteria remain the cornerstone of the nodal assessment, the limited diagnostic accuracy has been increased by the association with morphological parameters; it can be expected that the combination with other advanced MRI techniques (e.g., Diffusion-weighted Imaging, DWI) or functional parameters will further improve the diagnostic performance.

## 5. Advanced MRI Techniques: Diffusion-Weighted Imaging (DWI)

Diffusion-weighted Imaging (DWI) is a modified T2W sequence with motion-sensitive gradients to detect Brownian movements of water molecules within the tissues (“diffusion”) [61,62]. The magnitude of these gradients is described by the “b-value”: the signal of freely moving (diffusing) water molecules decays at increasing b-values; conversely, at high b-values, the signal from the water molecules with a restricted diffusion is significantly more intense [62]. Therefore, assuming that cellular membranes prevent water diffusion, the signal decay in DWI images is an indirect parameter of cellular density [63]. The relevance of DWI in oncological imaging is in the potential capability to provide information about increased cellularity, such as in malignant conditions [64,65], or reduced cellularity, such as response to chemotherapy [66,67], without any radiation exposure or administration of contrast material [68,69,70,71,72]. However, since the DWI sequence is highly prone to artifacts (e.g., T2 shine-through or susceptibility), it is recommended to evaluate the DWI images together with the apparent diffusion coefficient (ADC): a high ADC value reflects high water diffusion [73,74]. DWI and ADC together allow for a qualitative, the former, and quantitative, the latter, assessment by applying a monoexponential model [63]. 

Although the role of DWI in the assessment of RC is controversial, it has been proven useful for the evaluation of EMVI [75]. However, there is no consensus between the American and European guidelines: the first recommends the use of DWI both in primary staging and in restaging since DWI improves the detection of small lesions, the latter recommends DWI evaluation only in the restaging [16,17]. 

LNs are themselves characterized by a high cellular density of the lymphoid tissue, which exhibits a typical restriction pattern in DWI and therefore making them more easily detectable on DWI than T2W images. It has been demonstrated that the evaluation of DWI images improves the detection of pelvic LN by 10–83% when compared to conventional morphological MRI images alone [76,77,78,79].

Conversely, the visual, qualitative characterization of LNs on DWI is challenging given the natural high cellular density, with a consistent risk of over-staging [76,80]. This aspect is particularly relevant in restaging after neoadjuvant treatments. As previously mentioned, after CRT the number of LN detectable in the T2W morphologic sequences decreases, and this has been confirmed at DWI imaging; however, the specificity of DWI for the residual nodes was low (14%) and their characterization was uncertain [81].

**Figure 2:**
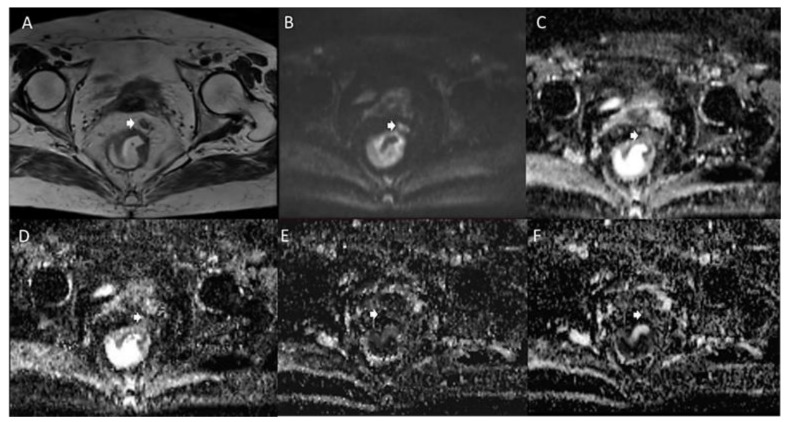
Woman 65 y.o. with rectal cancer. Tumor deposit (arrow) assessed in T2W sequence (**A**), in b 800 s/mm^2^ (**B**), in ADC map (**C**), in Dt map (**D**), in Dp map (**E**) and Fp map (**F**).

**Figure 3:**
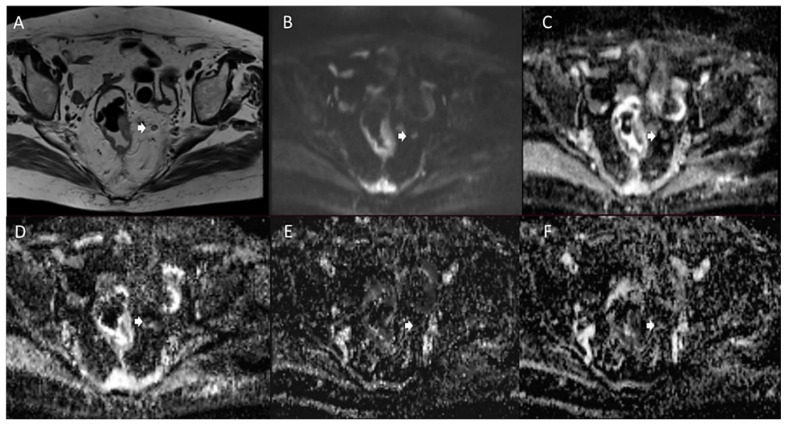
Man 72 y.o. with rectal cancer. Nodal assessment (arrow) in T2W sequence (**A**), in b 800 s/mm^2^ (**B**), in ADC map (**C**), in Dt map (**D**), in Dp map (**E**) and Fp map (**F**).

Many efforts have been done to pursue a quantitative and reproducible analysis of LNs with ADC values. High ADC values (low cellular density) correlate with benign LN; however, retrospectively determined thresholds showed unsatisfying sensitivities and specificities, ranging from 67–88% and 60–97%, respectively [77,82,83,84,85]. Moreover, the reproducibility of measurements can be influenced by the suboptimal resolution of ADC maps. Two studies reported that ADC could not be measured in a subset of 21–27% of the visible LN due to their small dimensions or local image distortions [76,77]; other studies excluded the LN <2mm because delineations were technically too challenging [84,85]. However, technical factors such as different scanner technology, b-values, different ADC calculations, and field strength represent the main drawback for the definition of an effective and robust threshold for ADC maps (Figure 2 and Figure 3) [86,87]. 

Chen et al. performed another kind of quantitative analysis. They evaluated the correlation between the tumor volume and the presence of lymphovascular invasion and LN metastases by comparing the measurements obtained on T2W images and on DWI [88]. Interestingly, the volume from DWI better correlated with the degree of invasion; this is probably related to the higher discriminative power of DWI than T2W images for the perirectal desmoplastic fibrotic reaction [88]. Additionally, the authors reported a threshold of 10.46 cm^3^ for the tumor volume identified on DWI that allows differentiation of N0 from N1–N2 with a sensitivity of 93.8% and specificity of 89.5%. However, these preliminary results need to be validated [88].

Therefore, even if DWI and ADC can provide promising results for LN staging in RC, the ESGAR panel recommends a qualitative assessment of these images given the lack of standardization, without a recognized role in daily practice [16]. 

## 6. Evolution of DWI Technique: Intravoxel Incoherent Motion (IVIM) and Non-Gaussian Model (Diffusion Kurtosis Imaging, DKI) 

As previously mentioned, DWI and ADC can describe the cellularity of the tissues. However, the monoexponential model does not consider the complex, non-Gaussian diffusion movements of the water molecules in vivo and is poorly accurate in the discrimination of the perfusion component of water (“pseudo-diffusion”) [89]. In the attempt of a more accurate model describing the physiologic and pathologic characteristics of tissues, other DWI models have been developed, namely the Intravoxel Incoherent Motion (IVIM) and Diffusion Kurtosis Imaging (DKI) [89].

IVIM is a bi-exponential model that separates the tissue diffusion and microcapillary perfusion by the application of the Levenberg–Marquardt nonlinear least-squares algorithm [90,91]. This allows for the extraction of a “pure” diffusion coefficient (D) for the water motion in the cellular interstitial space, and two perfusion-related parameters: pseudo-diffusion coefficient (D*), and perfusion fraction (*f*). D* considers the blood microscopic flow within the capillary vessels and *f* is the percentage of the water volume detectable within the capillary network; both are positively related to tissue perfusion [90,91]. These IVIM parameters could provide potential biomarkers of neoangiogenesis without the need for dynamic, contrast-enhanced studies [92,93]. 

In RC, several studies found significant correlations between IVIM-parameters and tumor pathology [94,95,96]. Regarding the LN evaluation, contradicting results were found [97,98,99]. Yu et al. analyzed the IVIM-parameters with LN dimensional features (short-axis diameter and short to long-axis diameter ratio) [97]. The D values resulted significantly lower for metastatic LNs, supporting that D is properly correlated to the diffusion of water within the tissues and that it is inversely correlated to cellularity [100]. Of note, the combination of short-axis diameter and D achieved higher AUC and sensitivity than any individual parameter. Malignant LN also showed lower D* values than the nonmetastatic nodes [97]. *f* values of malignant nodes were lower than non-metastatic LN as well, but the difference was not statistically significant. However, both the perfusion parameters were lower suggesting that, as in other tumors, malignant LN has reduced perfusion values [101]. Long et al. evaluated the IVIM-parameters on LN with three different short axes (SA, 3 mm ≤ SA ≤ 5 mm, 5 mm < SA ≤ 7 mm, and SA > 7 mm), reporting significantly lower D values in the metastatic LNs of 3 mm ≤ SA ≤ 5 mm, with no differences in the other groups [99]. The explanation of these results may lie in the greater necrotic component of bigger LN with associated higher diffusivity of water molecules. Moreover, the high cellularity (lymphocytes and plasma cells) of reactive LN could not be distinguished from LN invaded by malignant cells in the larger LNs groups. The authors did not find any statistical difference in D* values between metastatic and nonmetastatic LNs in all three groups [99]. However, *f* demonstrated lower values in 5 mm < SA ≤ 7 mm metastatic LNs compared to the nonmetastatic ones [99]. This might be due to the perfusion changes at different tumor growth levels, with a neoangiogenesis rate probably lower in the early phase of tumor growth. Qiu et al. confirmed that D* values are lower in metastatic LN, but in contrast to the other studies, they reported higher mean D, *f*, and ADC values in metastatic LN and concluded that average D and ADC values were more sensitive than *f* and D* values [98]. 

Contradicting results for D* and *f* values are reported also for other tumors [102,103,104], this could be partially due to incomplete knowledge of the pathophysiology of the carcinogenesis, the differences among MRI protocols, and the tissue characteristics within a magnetic field (echo time, relaxation kinetics) [105,106]; therefore, IVIM is currently not considered reliable in discriminating benign and malignant lesions.

**Figure 4:**
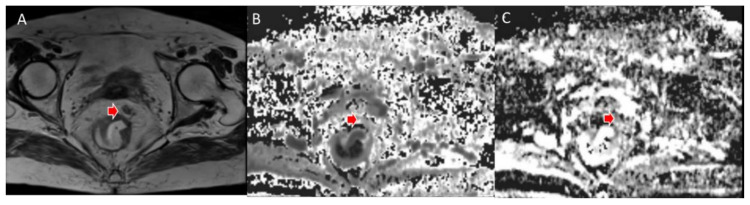
Woman 65 y.o. with rectal cancer (same patient as in Figure 2). Tumor deposit (arrow) assessed in T2W sequence (**A**), in MK map (**B**) and MD map (**C**).

**Figure 5:**
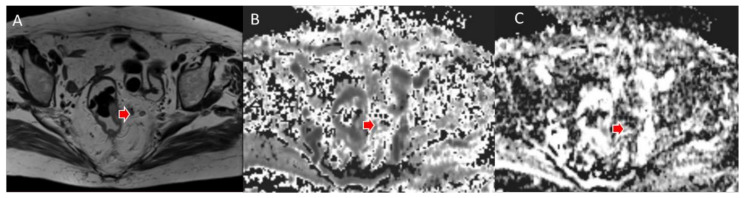
Man 72 y.o. with rectal cancer (same patient as in Figure 3). Nodal assessment (arrow) in T2W sequence (**A**), in MK map (**B**) and MD map (**C**).

DKI is based on a kurtosis model that considers the non-Gaussian movements of water diffusion and provides two coefficients: a dimensionless parameter that measures the deviation of the tissue diffusion from the Gaussian model, the K kurtosis coefficient (K), and a corrected ADC related to the non-Gaussian behavior, the diffusion coefficient (D) [107]. This approach already provided promising results in oncologic imaging, being more accurate than the traditional ADC maps in the diagnosis and tumor grading [108,109,110,111,112,113,114]. Specifically, in RC, DKI provided better performance in the assessment of tumor grade and treatment response, in the assessment of mucinous histotype and distant metastases [115,116,117,118,119]. DKI provided promising results also for the nodal assessment in RC. Zhou et al. segmented the rectal lesions on ADC maps: DKI parameters significantly correlated with N-stage (N0 vs. N1-2) [116]; Cui et al. reported similar results [120]. Yu et al. specifically analyzed the quantitative DKI values obtained from LN segmentation. Among other measurements, the median apparent diffusion of Gaussian distribution (Dapp) of metastatic LN was significantly higher than that of benign LN, with the highest AUC of 0.774. Moreover, the authors provided a threshold of 1126.15 × 10^−6^ mm^2^ s^−1^ with a sensitivity and specificity of 96.97% and 41.82%, respectively [121]. Therefore, the use of DKI in patients with RC is a promising technique that could improve the ability to predict the presence of metastatic LN in RC; however, further studies are necessary to standardize the technique (Figure 4 and Figure 5) [90].

Recently, a prospective study investigated the potential application of multiple DWI models, including IVIM and DKI, for nodal staging in patients without apparent nodal involvement on preoperative MRI [122]. The authors performed the analysis on the whole tumor and reported that RC with higher D* and DKI mean kurtosis values significantly correlated with the presence of LN involvement (pN1-N2). This potentially reflects the fact that tumors with higher microvascular perfusion and a more heterogeneous structure, as for more aggressive tumors, are more prone to nodal micrometastases. Again, the authors did not find any significant differences in *f* values, confirming the previous results [96,97]. However, they also performed a further analysis by combining some of the diffusion parameters and found that the combination of more DWI parameters improves the detection of micronodal involvement [122]. This promising evidence advises that in the future, multiple DWI models could be used in the evaluation of smaller LNs and micronodal involvement, which are the most challenging to be assessed with both morphological and functional MRI acquisitions. 

However, it must be pointed out that the time-consuming calculations to extract DWI data is a significant limit. Even if these are promising techniques, further studies are required to optimize the acquisition protocol by shortening the scan time, preserving the image quality, and identifying the optimal multiple b-values for the DWI, IVIM, and DKI studies in RC.

## 7. Dynamic Contrast-Enhanced MRI (DCE-MRI): The Role of Perfusion

Dynamic Contrast-Enhanced MRI (DCE-MRI) is a technique that evaluates tissue perfusion by the acquisition of multiple, sequential T1-weighted (T1W) images of an organ of interest after contrast medium administration [123]. Since angiogenesis correlates with tumor growth and spread, DCE gained a growing interest and its applications have been extended to all the body districts [124,125,126,127]; however, its value still has to be fully demonstrated [128,129,130]. 

**Figure 6:**
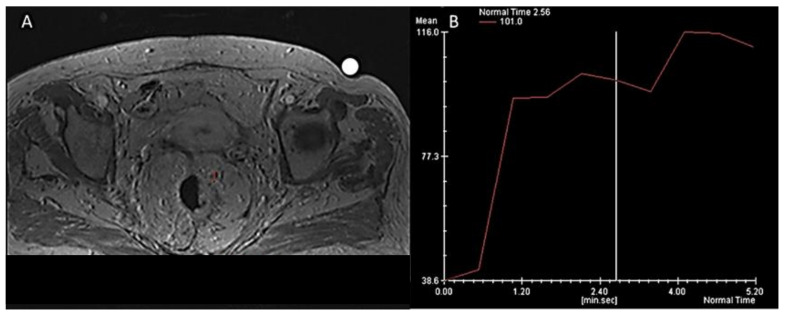
Woman 65 y.o. with rectal cancer (same patient of Figure 2 and Figure 4). DCE- MRI (**A**) deposit assessment with Intensity/Time curve evaluation (**B**).

**Figure 7:**
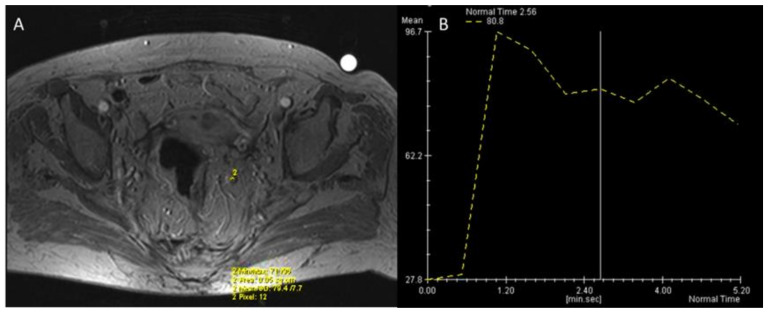
Man 72 y.o. with rectal cancer (same patient as in Figure 3 and Figure 5). DCE- MRI (**A**) nodal assessment with Intensity/Time curve evaluation (**B**).

In clinical practice, the most intuitive method to evaluate DCE-MRI is the qualitative, visual assessment of the time-intensity curves (TIC) describing the distribution of contrast material. Besides the differences among anatomical districts, usually up to four patterns of TIC are described [131,132,133]. This approach demonstrated a good reproducibility between the readers and satisfactory results in the assessment of breast lesions [134,135,136]; in RC, promising results have been reported in the detection of small tumors and the evaluation of treatment response [137,138,139,140]. The typical enhancement pattern of malignant LNs is an intense peripheral rim due to an active neoangiogenesis and a hypointense core, depending on the necrosis for the rapid tumor growth. This finding has been described by Alberda et al. in 51 patients after a long course of CRT in locally advanced RC [141]. An early and incomplete arterial enhancement on DCE provided an accuracy, respectively, of 93% and 89% for the diagnosis of positive LNs, with an optimal agreement between the two readers [141]. However, although simple and quick, the visual assessment is influenced by the radiologist’s experience and lack of reproducibility. In an afford to provide a more reproducible tool, many quantitative parameters have been proposed with various combinations of measurements [142,143,144]. Semi-quantitative perfusion maps provided encouraging results in RC treatment response and the assessment of malignant nodes with good reliability and reproducibility (Figure 6 and Figure 7) [145,146,147,148]. In a prospective study on 22 patients with locally advanced rectal cancer, semi-quantitative perfusion maps including the blood flow, the volume of distribution, and the mean transit time increased the sensitivity (71 to 86%) and specificity (70 to 90%) of conventional MRI sequences in the identification of mesorectal nodal metastases ≥ 5 mm [146]. 

Among the parameters extracted from perfusion models, the Ktrans is the most widely used: it is defined as the efflux rate of gadolinium contrast from the blood into the tissue extravascular extracellular space and is a marker of capillary permeability [124]. Grovik et al. found a correlation between a low Ktrans of the primary tumor and nodal metastases in 17 patients with resectable RC [147], while Yu et al. highlighted a significant correlation of Ktrans with malignant nodes only in a subgroup with diameter > 5 mm [148].

Another anatomical field of research is the mesorectum, directly involved in the tumor spread. Yoon et al. proposed the mesorectum fat area (MFA) as an independent prognostic factor of disease-free survival rate in patients with middle or low RC [149]. Recently, it became clear that the mesorectum affected by tumor diffusion also undergoes significant changes in micro/macro-vascularization, with a higher vessel diameter and denser branching compared to normal tissue [150]. Yang et al. aimed to assess whether quantitative DCE-MRI could detect these modifications. The authors observed lower Ktrans values of the tumor-surrounding mesorectum in the presence of malignant LNs compared with those with benign LN. These results, apparently conflicting with a conspicuous neoangiogenesis, can be related to the abnormal, intermitted, or interrupted, flow within the newly formed vessels, due to vessel occlusion by the tumor cells, with resulting low Ktrans values [151].

Some studies also reported better diagnostic performance of Ktrans compared to perfusion-related IVIM parameters, so DCE-MRI could eventually replace the time-consuming calculations of diffusion-weighted MRI parameters (such as the IVIM parameters D and D*); however, the administration of contrast material is necessary for DCE [152]. 

Even though the DCE-MRI has provided several encouraging results, the quantitative analysis showed limited reproducibility due to the many models applied [153,154]. Moreover, the problem of micro-metastases remains unresolved; this could be addressed with the identification of early changes in the tumor microenvironment, including the mesorectum. In the future, the key would probably rely on the correct combination of more parameters as already demonstrated for other pelvic tumors [155].

The issues discussed above limit the application of DCE-MRI for the routine assessment of the N-stage in the RC and there is still no consensus between the European and US guidelines [16,17]. Currently, the ESGAR panel considers contrast agent administration only in two clinical settings, specifically the assessment of tumor conspicuity after neoadjuvant treatment and the evaluation of mucinous tumors [16,156,157,158]. Conversely, the intravenous contrast agent is highly recommended by the guideline of the American College of Radiology (Society of Abdominal Radiology, SAR) [17]. Further studies are required to achieve the univocal consensus for the clinical application of DCE-MRI.

## 8. Positron Emission Tomography (PET): PET/CT and PET/MRI

PET/CT with 18F-fluorodeoxyglucose (FDG), which is part of the routine workup of advanced RC, has a high specificity and a low sensitivity for the detection of nodal metastases: inflammatory processes or the venous plexus can cause false-positive results; thus, the increased uptake is not a fully reliable diagnostic tool [159,160]. Therefore, other biomarkers, such as peak standardized uptake values (SUVmax and SUVpeak) and metabolic tumor volume (MTV) have been introduced as predictors of LN involvement and survival in several different tumors [161,162]. 

In RC, the MTV has been proposed as a biomarker predictive of survival in patients [163,164]; the MTV of the LNs improves the detection of nodal metastases [165,166]. Recently, Kim et al. obtained better results combining the nodal MTV with their SUVmax, with a specificity of 93.9% [167]. However, the sensitivity remained relatively low (48.5%) and similar to that of previous studies [159,168]. 

A relevant limitation of PET/CT is the poor spatial and contrast resolution of soft tissues, which makes it impossible to assess mesorectal LN with a diameter of less than 5 mm (below the resolution of PET). In addition, blooming artifacts from the uptake of the primary lesions may obscure adjacent uptake in small LNs [169]. These drawbacks could be overcome by PET/MRI. 

The synchronous observation of FGD uptake combined with the optimal contrast of soft tissues in PET/MRI makes this technique particularly helpful for the characterization of small, abnormal LNs. Catalano et al. observed a statically superior sensitivity of PET/MRI to MRI alone (79% vs. 58%) for the assessment of N-stage at baseline in a cohort of 62 patients with RC [170]. In PET/MRI workflow, the MRI sequences represent the time-consuming step of the examination. The associated, longer PET acquisition times (e.g., from 3 to 15 min) have higher event counts and signal-to-noise ratio, resulting in an increased number of detected LNs, in particular ≤5 mm, at the same examination time [171]. However, a clear threshold of SUV values of small perirectal LNs has not been defined because of the small dimensions and partial volume artifacts: a nodal uptake greater than the background is considered positive [172]. Crimì et al. examined the restaging of 36 patients with locally advanced rectal cancer after CRT, highlighting a slightly higher accuracy in T (92% vs. 89%) and N staging (92% vs. 86%) for whole-body FDG PET/MRI than for MRI alone. PET/MRI findings also prompted changes to the treatment strategy in 11% of cases when hypermetabolic tumor residuals were detected within the areas of fibrosis [173]. In contrast, Kang et al. observed the same overall accuracy (41.7%) of CT and PET/MRI for the N category [174]. 

In conclusion, the limitations of PET/CT and MRI in the nodal assessment can be partially overcome by PET/MRI.

## 9. Radiomics: Images Are Data

Radiomics has the purpose to provide clinical information from the extraction of quantitative data (features) from medical images [175,176]. Thanks to Artificial Intelligence (AI), hundreds of radiomics features are extracted from a region/volume of interest (ROI/VOI) and are evaluated by high-order statistical analysis with Machine Learning (ML) and Deep-Learning (DL) to be correlated to the clinical outcome [177,178,179,180]. In the era of precision medicine, a post-processing quantitative technique potentially able to support decision-making in different clinical settings is particularly appealing [181]. Therefore, in the last decade, plenty of papers have been published, mostly in oncological imaging and with different imaging techniques, reporting the potential role of radiomics in diagnosis, characterization, and evaluation of the tumor response to treatments [182,183,184,185] and nodal assessment [186,187,188]. 

The easiest level of radiomics is texture analysis: it evaluates the distribution and relationships of the pixels/voxels. It is an active field of research in oncological imaging [189,190] and preliminary results are available in the assessment of RC [191,192]. Texture analysis provides different orders of features that are clustered by the number of “statistical steps” required to extract them. Histogram parameters represent the first order: the pixel values are analyzed without considering their relationships [193]. Preliminary data showed the value of histogram analysis as a quantitative analysis tool for MRI (DCE, DWI) in RC [194,195]. Liu et al. performed a histogram analysis on the ADC map of the whole tumor and reported that entropy was an independent predictor of nodal involvement [196]. Recently, Yang et al. performed the same analysis on T2W of the whole tumor: they found that a lower skewness was an independent risk factor for LN metastases (odds ratio 9.832; 95% CI, 1.171–56.295; *p* = 0.01); moreover, a difference in entropy was reported, but it was not an independent predictor of nodal involvement [197]. As in several clinical settings, different parameters are combined into nomograms depending on the respective relevance in the decision process, an additional step in radiomics analysis will be the development of prediction models including multiple features in the so-called “radiomics signature”. This new approach is providing preliminary, significant results in risk stratification of different tumors and from both CT and MRI images [198,199]. 

For this purpose, Huang et al. developed a radiomics normogram that integrated the LN qualitative evaluation on CT, carcinoembryonic antigen (CEA), and a radiomic signature obtained from a region-of-interest (ROI) of the whole tumor on the portal phase of CT. The authors reported that this normogram successfully stratified patients according to their risk of LN metastases achieving a C-index of 0.74 in the training set and 0.78 in the validation set [200]. Chen et al. went further, as they proposed a multi-modality radiomics signature for nodal assessment that integrates the advantages of different imaging modalities: contrast-enhanced CT (blood flow information) and endorectal ultrasound with shear-wave elastography (stiffness). This multiparametric model obtained a higher performance (c-index of 0.87 in the training set and 0.86 in the validation set) when compared to the conventional nomogram based on enhancement changes of the tumor [201].

Predictive models with radiomics signatures have been developed also for the nodal assessment in MRI (T2W, DWI/ADC). In a recent retrospective single-center study, radiomics features were extracted from preoperative high-resolution T2WI of different histological RC and analyzed using different algorithms. The random forest analysis showed a good diagnostic performance for the N-stage with an AUC of 0.746. The prediction model was able to differentiate N0 from N1-N2 patients with a sensitivity of 79% and a specificity of 72% [202]. Similar results (81% sensitivity and 68% specificity) have been recorded with a model derived from DWI radiomics features; the segmentation was performed on the primary tumor [203]. Zhu et al. compared the performance of two models based, respectively, on the radiomics signature of the primary tumor and of the LNs, before and after CRT, for the prediction of nodal involvement in advanced RC [204]. The authors concluded that the features from the LN model perform better than the tumor features for the prediction of nodal involvement [204]. Moreover, when compared to the radiologist, the radiomics model had higher specificity (60% vs. 43%), while sensibility was similar (95% vs. 100%) [204]. Subsequently, Li et al. performed a radiomics analysis on LN with a short axis ≥ 3 mm in the mesorectal (peritumoral) or superior mesenteric region from morphologic T2W images. The authors compared the subjective assessment of the radiologist with the radiomic model; the latter demonstrated better diagnostic performance, with sensitivity, specificity, and accuracy, respectively, of 92.23%, 84.69%, and 89.88% [205]. This point has been also investigated by Zhou et al. who evaluated a multi-parametric MRI radiomics model for nodal assessment following CRT by combining the radiomic signature with an experienced radiologist’s visual evaluation. The integrated model improved the negative predictive value (NPV) from 92.2% to 93.7%; in particular, the NPV was 100% in the yT1-2 subgroup after CRT [206]. These data suggest that an integrated approach provides better decision-making models even when different techniques are combined [207]; further studies are required for RC.

The main drawback of radiomics is reproducibility: different scanners, acquisition protocols, image reconstruction, or ROI segmentation methods have an impact on the extracted features [208,209]. Regarding MRI in RC, texture features are not significantly different across magnetic field strengths (1.5T vs. 3T) [210], and radiomics models independent from the field strength are being developed [211]. Some studies also reported that first-order textural features and fractal parameters have higher repeatability than high-order parameters [212]. About CT in RC, textural features obtained from contrast and non-contrast-enhanced image are not equivalent [213]. Therefore, more data regarding repeatability are necessary to enhance the accuracy of radiomics models to allow their application in the clinical scenario [214,215].

## 10. Experimental Applications: Ultrasmall Superparamagnetic Iron Oxide (USPIO)

Ultrasmall superparamagnetic iron oxide (USPIO) nanoparticles are MR contrast agents developed for the evaluation of the lymphatic system [216]. When administered intravenously, USPIOs are absorbed by the normal reticuloendothelial elements, including nodal macrophages, distributed within the medullary sinus. Briefly, the superparamagnetic effect of USPIOs generates in normal LN a loss of signal on T2W and T2*W called ‘susceptibility effects’, whereas depleted phagocytosis of the malignant LNs leads to an increase in SI [33,217]. Therefore, the nodal assessment could be performed on cellular constituents rather than morphology or size. 

A meta-analysis by Will et al. reported a pooled sensitivity of 88% and a specificity of 96% in the nodal assessment of various kinds of tumors [218]. Four different patterns of SI for mesorectal LN in RC were reported. All non-malignant LN showed uniform or central low SI patterns, while eccentric and uniform high SI patterns were noted in metastatic LNs [216]. A higher frequency of the central low signal in reactive LN is also reported, opening the possibility of using USPIO in doubtful cases to reduce the false positives of MRI [216]. Further validation of these data is necessary since the study included 12 patients with resectable tumors and LNs ≥3 mm [216]. The issue of LN size has been reported also for other body districts, being 5 mm the lowest dimensional limit [219]. 

Assuming that the outcomes of previous studies are related to the possible lower resolution on 1.5 T scans, Stijns et al. evaluated the diagnostic performance of USPIO in RC at a sub-millimeter isotropic resolution on a 3T MRI scan. Unfortunately, out of the 55 LNs characterized as pathological, only six were metastatic at the node-to-node analysis, with a low true-positive rate (11%). Additionally, 20 false positives LN with high SI on USPIO-enhanced MRI were reactive at pathology [220]. 

It has to be pointed out that USPIO is approved only for the treatment of anemias and shows some relevant adverse effects [221]. Therefore, even if interesting, the application in the routine diagnostic workup of RC is not feasible.

## 11. Advanced CT Techniques: Dual Energy CT (DECT)

In recent years, dual-energy computed tomography (DECT) has been widely employed in clinical practice [222,223]. DECT can overcome the limits of conventional CT for soft tissue resolution, combining morphologic and functional information [224,225,226,227].

Few preliminary studies reported a significant correlation of DECT quantitative parameters with LN metastasis [228,229]. Liu et al. tried to predict LN involvement in RC by matching functional and dimensional parameters of regional LN. The authors calculated the normalized iodine concentration (nIC), which is the ratio between the iodine concentration of the LN and the iliac artery (nIC = IC LN/IC artery), both on arterial and venous DECT datasets. When the nIC from the portal phase was combined with the dimensional criteria, the authors reported the highest accuracy for detection of metastatic LN (Sensitivity 75.6%; Specificity 88.3%; Accuracy 82.9%) [228]. Similarly, Sato et al. evaluated the role of nIC in enlarged pararectal and lateral LN for low RC. They partially confirmed the previous results: in pararectal LN the authors found significant differences in nIC, but not in dimensional parameters. Conversely, in lateral LN, the authors found significant differences in dimensional parameters and nIC from the venous DECT dataset [229].

Despite the promising results in functional LN assessment, the diagnostic advantages of DECT over MRI are not yet established [230]. Therefore, considering the radiation exposure and the poor diagnostic performance, DECT is not currently indicated in the nodal assessment of RC patients [231]. MRI remains the reference standard, despite the already discussed advantages and weaknesses [56].

## 12. Reporting

Detailed and consistent reporting is critical for accurate and effective communication among multiple disciplines; therefore, structured reporting is usually recommended [232,233,234]. Since the overall management of RC in the United States and Europe is different, the recommendations for LN assessment and reporting are different in ESGAR and SAR guidelines [16,17]. 

Regarding LN assessment in primary staging (please refer to Table 1 for classification of nodal stations), ESGAR recommends the combination of size and morphological criteria, whereas in SAR guidelines the dimensional criteria did not obtain univocal consensus. Specifically, suspicious LNs have a round shape, irregular border, or heterogeneous SI on MRI [16,17]. According to the 2016 ESGAR guidelines, a LN is classified as metastatic when it shows: (a) a short-axis diameter greater than or equal to 9 mm; (b) a short-axis diameter of 5–8 mm and two or more morphologically suspicious characteristics (round shape, irregular border, and/or heterogeneous signal); (c) short-axis diameter of less than 5 mm and three morphologically suspicious characteristics; and (d) a mucinous content (any size) [16]. The radiologist is invited to specify the number of LN and their location, whether mesorectal or extra-mesorectal, and to report the presence of any deposits within the mesorectum. From a practical point of view, the extra-mesorectal nodes are classified in the same way, but there are still no specific standards for the assessment of these LNs.

It has to be remembered that internal iliac and obturator LNs are outside the CRM and that the presence of “high” LNs (eg. principal IMA LN) has an impact on the upper borders of the radiotherapy volume, therefore it a proper assessment and reporting of these nodal groups is fundamental [22,34].

A notable difference between SAR and ESGAR guidelines is in LN restaging. North American guidelines consider that nodal downsizing after CRT is itself a sign of disease eradication, while the European panel advises that treated nodes with a short-axis > 5 mm should be assessed as malignant, despite notable dimensional reduction [235].

All this information leads to the definition of the structured report based on the key radiological findings that are necessary for the proper decision of the treatment strategy, both at baseline or at restaging after CRT [236]; the structured report is highly recommended because of significant improvements in radiological workflow [237,238].

## 13. Conclusions

Currently, MRI remains the main imaging modality recommended for LN evaluation in RC. However, the radiologist should be aware of the low specificity of MRI in the detection of lymph node metastases even when combining size with other parameters (e.g., DWI/ADC).

Many research efforts have provided promising results in different fields, such as MRI and CT advanced techniques (e.g., IVIM, DKI, DCE-MRI, USPIO, DECT), hybrid imaging (e.g., PET/CT, PET/MRI), and image analysis (i.e., Radiomics). However, a significant proportion of the parameters developed are still far from clinical routine: a possible reason relies in the incomplete pathophysiological comprehension of nodal involvement in RC. In the next future, a probable winning strategy will not be the research of a single “best” imaging modality or parameter (e.g., a single threshold value or a radiomic feature), but the integration of more imaging parameters, even with clinical and pathological data. This strategy would be actually feasible during this developing era of AI: this tool could be the key toward a more precise assessment of the LN involvement in RC.

## Figures and Tables

**Table 1:** Lymph nodes compartments and anatomical boundaries in N-stage assessment as for the AJCC-TNM 8th edition.

Compartments				Boundaries and Considerations		TNM
Mesenteric	Pararectal/mesorectal LN			Within the mesorectum	The most common pathway of nodal spread (mostly tumors above the PR)	N
	Superior rectal LN			At the level of the superior rectal A		N
	IMA LN			Between the origin of the left colic artery and immediately below the origin of the IMA		N
	Principal IMA LN			Origin of the IMA		N
Extra mesenteric	Pelvic sidewall LN	Internal iliac/hypogastric LN		Along the hypogastric A	Frequently involved if the tumor is at or/and below the PR (NB outside of the CRM)	N
		External iliac LN	Lateral chain	Lateral to the external iliac A it continues in the lateral chain of the common iliac LN	Rarely involved; could be involved if the tumors are at and below the PR or exceptionally in tumor extending below the dentate line (through superficial inguinal LN)	M
			Middle chain	Between the external iliac A and V		M
			Medial chain	Posterior to the external iliac V	Could be involved if the tumors are at and below the PR Frequently indistinguishable from obturator LN (i.e., along the obturator A), which are frequently involved as well	M
		Common iliac LN	Lateral chain	A continuation of the lateral chain of the external iliac LN	Could be involved if the tumors are at and below the PR	M
			Medial chain	Between the common iliac A at the sacral promontory		M
			Middle chain	A continuation of the hypogastric/internal iliac region and the lateral sacral region. Sited posteriorly to the common iliac A and V, abutting the L5 nerve root as it passes anterior to the sacral alae		M
	Retroperitoneal LN	Left para-aortic		To the left of Aorta		M
		Right latero-aortic		Aortocaval, precaval, laterocaval, and retrocaval		M

Legend: LN, lymph nodes; A, artery/arteries; V, vein/veins; PR, Peritoneal Reflection; AJCC-TNM, American Joint Committee on Cancer-Tumor-Node-Metastasis classification; CRM, circumferential resection margin; IMA, inferior mesenteric artery; AJCC, American Joint Committee on Cancer Commission.

## Data Availability

Not applicable.
